# Hospital Life in Macedonia

**Published:** 1918-04-20

**Authors:** Private P. A. Lawrence

**Affiliations:** R.A.M.C.


### HOSPITAL LIFE IN MACEDONIA.

By PRIVATE P. A. LAWRENCE, R.A.M.C.

We are indebted to the *Birmingham Post* for the following extracts from an article by Private Lawrence on life in a general hospital in Macedonia. The manuscript was sent to the Rev. E. H. Manning, chaplain to the Birmingham General Cemetery, and was forwarded by him to the Midland journal:

"I have never heard of a book dealing with the conditions of living and the details of the work of the soldier engaged in hospital work in the field. There is, for instance, an opening for a skilful someone to paint a series of word-pictures as seen in the every-day routine of a general hospital, and a particularly fine opportunity might be afforded if such a one centred his attention on a general hospital here.

"Compared with units of a similar order elsewhere the hospital has a distinctive claim on the general interest. Perhaps the chief of these is, though manned by British R.A.M.C. *personnel*, it is attached for duty to the Royal Serbian Army, and its patients are all Serbs.

"The hospital left England in August, 1916, ostensibly bound for Egypt. But actually our destination was in the Salonika region, and somewhere in the Balkans we pitched our camp, and here we have remained. At present it strikes the stranger for the most part as a great dreary waste of land, which the eyes of the most covetous land-grabber might very well avoid. Here for a year and a half we have lived and moved and had our being. We haven't been in the limelight. On the other hand, we have experienced the drudgery of a humdrum routine, with an aloofness from the main current of the world, virtually isolated from all movements even in this particular zone of war. We have had to contend with the treachery of the Macedonian climate, and, what is perhaps worst of all, with the grim and threatening spectre of disease.

"It isn't easy for fellows of spirit, and sometimes refinement, to pursue menial tasks day after day. It isn't easy to endure a virtual severance from the world, to be restricted in one's correspondence to the writing of two letters each week, and to have to wait, and wait, and wait for mails, mails that are so often delayed, and mails that sometimes never arrive, as, for instance, our recent Christmas parcel mail. It isn't easy to live in a land of strange people. It isn't easy to live in a neighbourhood where the deadly mosquito holds an almost undisputed sway in the summer season. It isn't easy to enjoy the vagaries of a climate where in summer we frizzle and in winter we freeze, and where the winds are gales or sandstorm blizzards. And it isn't easy to avoid the deadly malaria and the no less deadly dysentery, where the enemy, if not a Bulgar, is a deadly bacilli; where, though he may not be a German, he is a powerful germ. Thus, in some respects at least, life is not a picnic in Macedonia.

"There is naturally plenty of grousing, but the really unhappy man is the exception. Laughter and good cheer prevail. Within the hospital many men in such spare time as is available redeem the time by football and cricket and other sports. Occasional concerts and whist drives are a relaxation. Christmas pantomimes have been produced on lines that would amaze the people at home, and that would even do credit to a first-class house in England. And the presentation of two first-class musical operas, splendidly set and rendered, with full scenic and musical accompaniment, has been a triumph. Meanwhile the purpose for which we were sent here is pursued day by day, and week by week."

